# Comparison of Magnesium and Titanium Doping on Material Properties and pH Sensing Performance on Sb_2_O_3_ Membranes in Electrolyte-Insulator-Semiconductor Structure

**DOI:** 10.3390/membranes12010025

**Published:** 2021-12-25

**Authors:** Chyuan-Haur Kao, Kuan-Lin Chen, Jun-Ru Chen, Shih-Ming Chen, Yaw-Wen Kuo, Ming-Ling Lee, Lukas Jyuhn-Hsiarn Lee, Hsiang Chen

**Affiliations:** 1 Department of Electronic Engineering, Chang Gung University, 259 Wen-Hwa 1st Road, Kwei-Shan District, Tao Yuan City 333, Taiwan; chkao@mail.cgu.edu.tw (C.-H.K.); klchen@mail.ncku.edu.tw (K.-L.C.); 2 Kidney Research Center, Department of Nephrology, Chang Gung Memorial Hospital, Chang Gung University, No. 5 Fuxing St., Guishan District, Taoyuan City 333, Taiwan; 3 Department of Electronic Engineering, Ming Chi University of Technology, 284 Gungjuan Rd., Taishan Dist., New Taipei City 243, Taiwan; 4 Department of Applied Materials and Optoelectronic Engineering, National Chi Nan University, Puli 545, Taiwan; s107328002@mail1.ncnu.edu.tw (J.-R.C.); s107328009@mail1.ncnu.edu.tw (S.-M.C.); 5 Department of Electrical Engineering, National Chi Nan University, Puli 545, Taiwan; ywkuo@ncnu.edu.tw; 6 Department of Electro-Optical Enginnering, Minghsin University of Science and Technology, No.1, Xinxing Rd., Xinfeng 304, Taiwan; 7 National Institute of Environmental Health Sciences, National Health Research Institutes, Zhunan 350, Taiwan; lukaslee@nhri.edu.tw

**Keywords:** Mg doping, Ti doping, pH sensing, Sb_2_O_3_, silicate, crystallization

## Abstract

In this research, electrolyte-insulator-semiconductor (EIS) capacitors with Sb_2_O_3_ sensing membranes were fabricated. The results indicate that Mg doping and Ti-doped Sb_2_O_3_ membranes with appropriate annealing had improved material quality and sensing performance. Multiple material characterizations and sensing measurements of Mg-doped and Ti doping on Sb_2_O_3_ sensing membranes were conducted, including of X-ray diffraction (XRD), X-ray photoelectron spectroscopy (XPS) and transmission electron microscopy (TEM). These detailed studies indicate that silicate and defects in the membrane could be suppressed by doping and annealing. Moreover, compactness enhancement, crystallization and grainization, which reinforced the surface sites on the membrane and boosted the sensing factor, could be achieved by doping and annealing. Among all of the samples, Mg doped membrane with annealing at 400 °C had the most preferable material properties and sensing behaviors. Mg-doped Sb_2_O_3_-based with appropriate annealing are promising for future industrial ionsensing devices and for possible integration with Sb_2_O_3_-based semiconductor devices.

## 1. Introduction

Over the past fifty years, growing attention has been paid to the development of the chemical sensing of ion concentrations in various solutions. Measurements of ion concentrations, such as pH sensing, are crucial to monitor human health, food safety and environmental pollution. Owing to rapid detection, fast response and reliable long-term operations, semiconductor-based ion sensitive devices have been proposed, such as ion-sensitive field-effect transistors (ISFETs), electrolyte-insulator -semiconductor (EIS) capacitors and light-addressable potentiometric sensors (LAPS-) [1,2]. Among these devices, EIS capacitors, with their advantages of compact size, low cost and simple fabrication, have been demonstrated as multianalyte ion and solute sensing devices [3,4]. In an EIS capacitor, the key component is the sensing membrane, capable of detecting ions in solutions. For conventional EIS devices, SiO_2_ is commonly used as the dielectric for the membrane, though materials such as Ta_2_O_5_ [5], Gd_2_O_3_ [6] and Zr_2_O_3_ have also been studied as possible replacements for traditional SiO_2_. In order to further boost the EIS capacitor’s performance and provide the possibility for future device integration, it is worthwhile to explore new materials and treatments for the membrane material in order to fabricate EIS capacitors with pH-sensing behaviors. Based on previous reports [7,8], Sb_2_O_3_ has been used for transparent conducting films, varistors and photocatalysts. In addition, the usage of Sb_2_O_3_ can improve electrical and optical device properties. Based on recent studies [9,10,11,12,13,14,15,16,17,18,19] Sb_2_O_3_ has been used for gas and liquid solution sensors. However, Sb_2_O_3_ EIS sensors have not been clearly reported yet. Compared with conventional semiconductor materials, Sb_2_O_3_ with a wide band gap of around 3 eV [20] can withstand high breakdown voltage. As for new treatments, incorporating various atoms such as F [21], N [22], Ti [23] and Mg [24] by plasma treatment [25] or co-sputtering [26] into EIS membranes to strengthen their material quality has been intensively investigated. For foreign atoms dopings, Ti doping and Mg doping via cosputtering can optimize the EIS sensor’s sensing behaviors because the incorporated atoms can passivate defects. Moreover, Mg doping could further boost the sensitivity and reliability of EIS sensors, owing to the decrease of the double-layer capacitance in the solution and sensing factor enhancement. Until now, however, comparison of the effects of Mg doping and Ti doping on EIS membranes has not been demonstrated. In this study, Mg-doped and Ti-doped Sb_2_O_3_ were fabricated as membranes with excellent sensing performance. However, EIS capacitors with Mg-doped or Ti-doped Sb_2_O_3_ [27,28,29,30,31,32,33,34,35] as the membrane material have not been clearly reported yet. Since Mg has low electron affinity and low electronegativity, and the radius of Mg^2+^ is similar to that of Sb^3+^, Mg can perfectly replace the lattice site of Sb [36,37,38,39,40,41,42,43]. In addition, many studies have also shown that Mg doping can increase the energy gap and reduce redundant oxygen vacancies. Moreover, annealing treatment in an O_2_ ambient can further improve sensing performance and device reliability. This is because filling the oxygen vacancy and reducing the defects in an oxygen environment repairs the dangling bonds and releases the strain bonds, while more oxygen atoms are added to the surface and the oxygen vacancies in the lattice are filled. Therefore, Mg-doped Sb_2_O_3_ EIS sensors with annealing at 400 °C can achieve a high sensitivity of 60.17 mV/pH, which is above the Nernst limit [44]. To gain insight into the effects of annealing, multiple material analyses including X-ray diffraction (XRD), X-ray photoelectron sptroscopy (XPS) and field-effect scanning electron microscopy (FESEM) were used to examine the surface morphologies and material properties of Sb_2_O_3_ membranes. Material characterizations reveal that annealing at an appropriate temperature can enhance Sb_2_O_3_ crystallization and Ti doping of the Sb_2_O_3,_ and Mg doped of the Sb_2_O_3_ can suppress the formation of silicate grainization. Therefore, the EIS pH-sensing capability could be boosted and reliability issues such as hysteresis and drift voltage can be mitigated. Mg-doped Sb_2_O_3_-based EIS capacitors are promising for versatile integration in chemical ion-sensing applications with other Sb_2_O_3_-based devices [45,46,47,48].

## 2. Experimental

**Figure 1:**
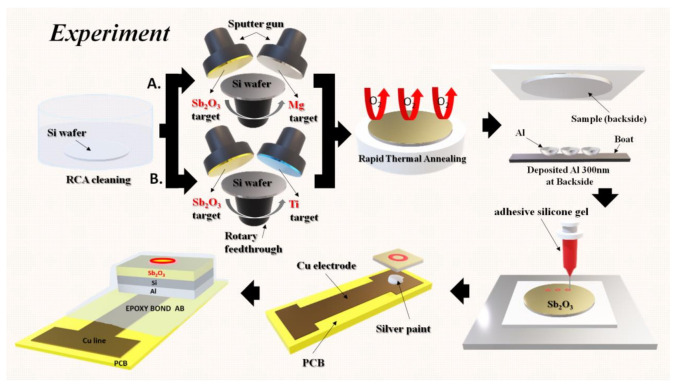
A diagram of the fabrication process including, RCA cleaning, co-sputtering, rapid thermal annealing, aluminum film backside deposition, sensing area definition and the attachment of the sensing chip onto the PCB board.

To fabricate Sb_2_O_3_-based EIS capacitors, the films were deposited on 4-inch n-type (100) wafers with a resistivity of 5–10 Ω-cm. The Sb_2_O_3_ was sputtered, and Mg or Ti was co-sputtered on the wafers in an Ar:O_2_ = 20:5 ambient, respectively. The RF power was l00 W and the pressure inside the chamber was l0 mTorr. Based on the measurements, the thickness of the undoped Sb_2_O_3_ was 61.53 nm, the thickness of Mg-doped Sb_2_O_3_ was 63.40 nm and the thickness of the Ti-doped Sb_2_O_3_ was 69.25 nm. The rapid thermal annealing (RTA) process was carried out for 30 s in O_2_ ambient at temperatures of 400, 500 and 600 °C, respectively. Then, a 300 nm aluminum film was deposited on the backside of the wafer. The backside aluminum film was grown by e-beam evaporation. After that, an epoxy bond was used to determine the sensing area. Finally, silver gel was used to attach samples on the copper wires of the printed circuit board (PCB). The detailed EIS structure is shown in Figure 1.

Analyses using CV measurements were performed. Using a reference capacitance of 0.4 C_max_ in the CV curves, the correlation between the substrate bias and the electrolyte concentration was calculated. In addition, the change of substrate bias voltage variation caused by the change of electrolyte concentration can be illustrated by the site-binding model [49,50], with the flat band voltage shift proportional to the electrolyte concentration, as in the following equation:

(1) $$V_{FB}=E_{Ref}-\Psi_{0}+\chi^{sol}-\frac{\Phi_{Si}}{զ}-\frac{Q_{ox}-Q_{ss}}{C_{ox}}$$

$E_{Ref}$ is the reference electrode potential, and $\chi^{sol}$ is the surface dipole potential of the solution. $\Phi_{Si}$ is the work function of silicon, and $\Psi_{0}$ is the liquid junction potential difference. All of the terms in the equation are constant, except for $\Psi_{0}$, which makes the membrane sensitive to the electrolyte due to polarization and forms the potential barrier. Furthermore, $\Psi_{0}$ is closely related to the surface sites on the membrane.

Moreover, the hydrogen ionic reaction with the membrane interface is illustrated in the site-binding model shown in Equation (2) [51,52,53]. The surface potential (*ψ*) can be related to the membrane parameter *β. k* is Boltzmann’s constant, *q* is the elementary charge, *T* is the temperature, *pH_pzc_* is the *pH* value with zero charge on the surface and *β* is a factor that points to the sensitivity of the gate membrane.

(2) $$\psi =2.303\frac{kT}{q}\frac{\beta }{\beta +1}({pH}_{pzc}-pH)$$

Furthermore, β is closely related to the density of surface hydroxyl groups, as shown in (3). N_s_ is the number of surface sites per unit surface area and *C_DL_* is the double layer capacitance, according to the Gouy–Chapman–Stern model [54].

(3) $$\beta =\frac{2q^{2}Ns\sqrt{K_{a}K_{b}}}{KTC_{DL}}$$

**Figure 2:**
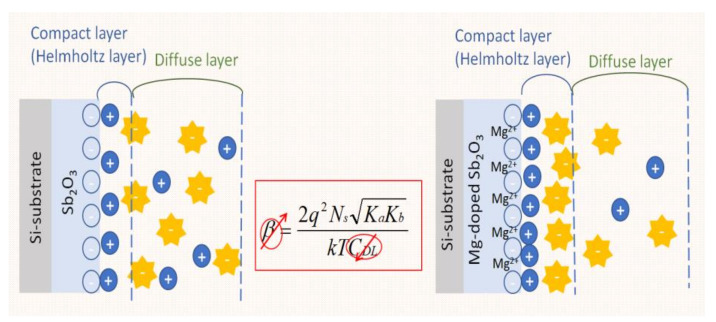
An illustration of the Mg co-sputtered on the Sb_2_O_3_-based EIS capacitor.

In this research, Mg-doped Sb_2_O_3_-based EIS capacitors were demonstrated as the best pH-sensing devices, as shown in Figure 2. The tighter inner layer becomes the Helmholtz layer, which causes the positive charge to be absorbed by the Helmholtz layer and is not affected by the potential difference.

Therefore, the outer layer is still a diffusion layer, and the resulting capacitance C_DL_ is reduced. It is known from the Moss-Berstein effect that, when the capacitance C_DL_ decreases, the β value increases, so, the higher β is, the better the sensing capability will be [55,56].

Based on (3), the higher the surface site density of N_s_ is, the higher β is and the better the sensing capability will be. The grainization, crystallization and compactness of the material structure on the sensing membrane may reinforce the quality and quantity of the surface sites.

## 3. Results and Discussion

**Figure 3:**
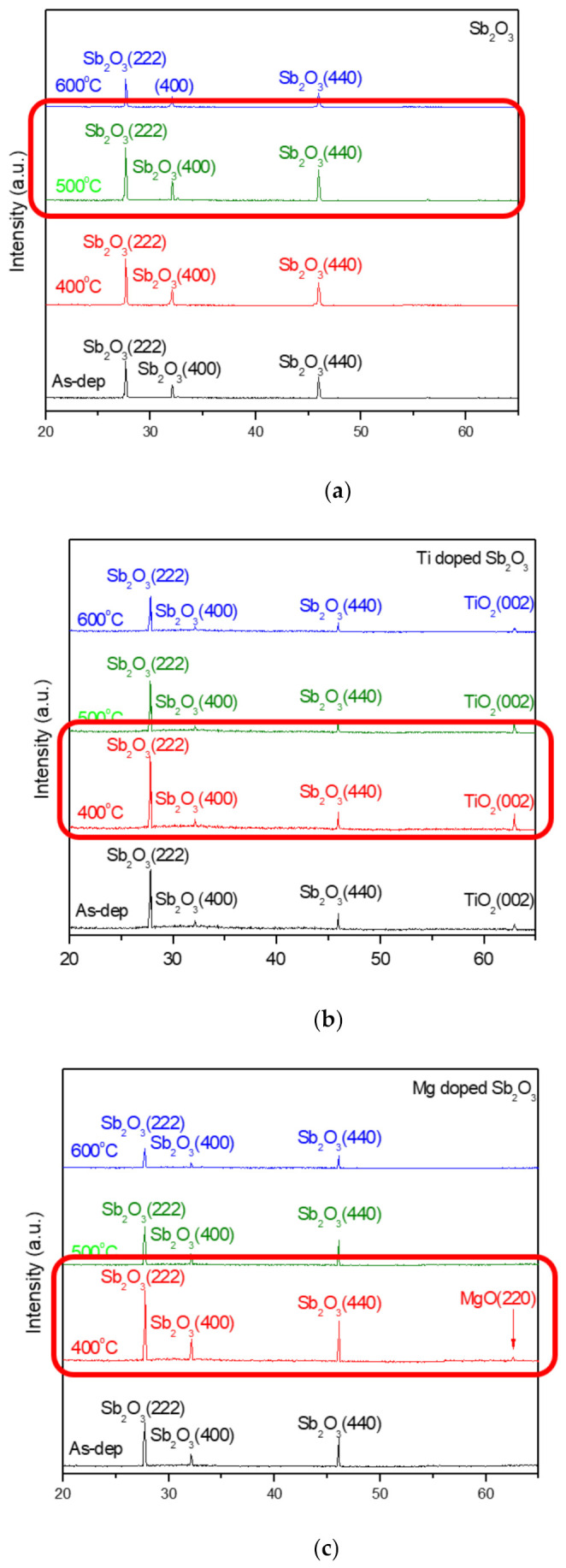
XRD patterns of (**a**) the undoped Sb_2_O_3_ films, (**b**) Ti-doped Sb_2_O_3_ films and (**c**) Mg-doped Sb_2_O_3_ films annealed at various temperatures in O_2_ ambient for 30 s.

To investigate the effects of Ti doping and Mg doping with annealing on a Sb_2_O_3_ membrane, XRD was used to monitor the crystalline phases of the differently treated films. The XRD patterns of the undoped, Ti-doped and Mg-doped samples are shown in Figure 3a–c, respectively. As shown in Figure 3a–c, Sb_2_O_3_ (222), (400) and (440) phases can be observed in the XRD patterns of all of the samples. Among the undoped samples, the film annealed at 500 °C had the strongest crystalline phases. In addition, among Ti-doped and Mg-doped samples, the samples annealed at 400 °C had the strongest crystallized phases. Furthermore, Ti-doped and Mg-doped samples had stronger XRD peaks than the undoped samples, indicating that the combination of doping could further enhance the crystallization. However, as the anneal temperature increased to 400 and 500 °C, all of the Sb_2_O_3_ peaks increased, indicative of the crystalline phases strengthening and the crystallization enhancing. As the annealing temperature increased to 600 °C, the Sb_2_O_3_ decreased, and the crystalline structures might be deteriorated in undoped, Ti-doped and Mg-doped samples owing to the RTA pH-sensing devices at 600 °C.

**Figure 4:**
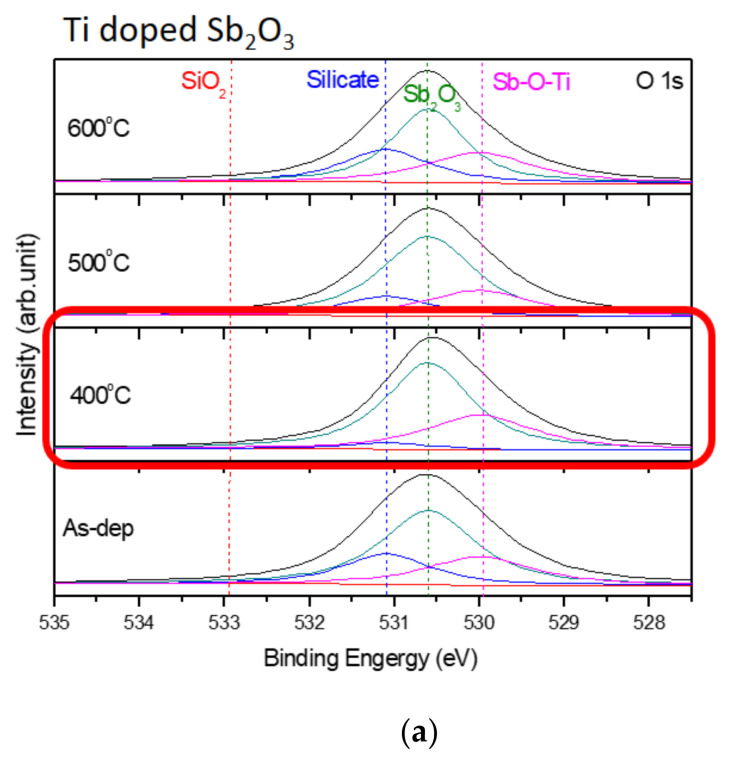

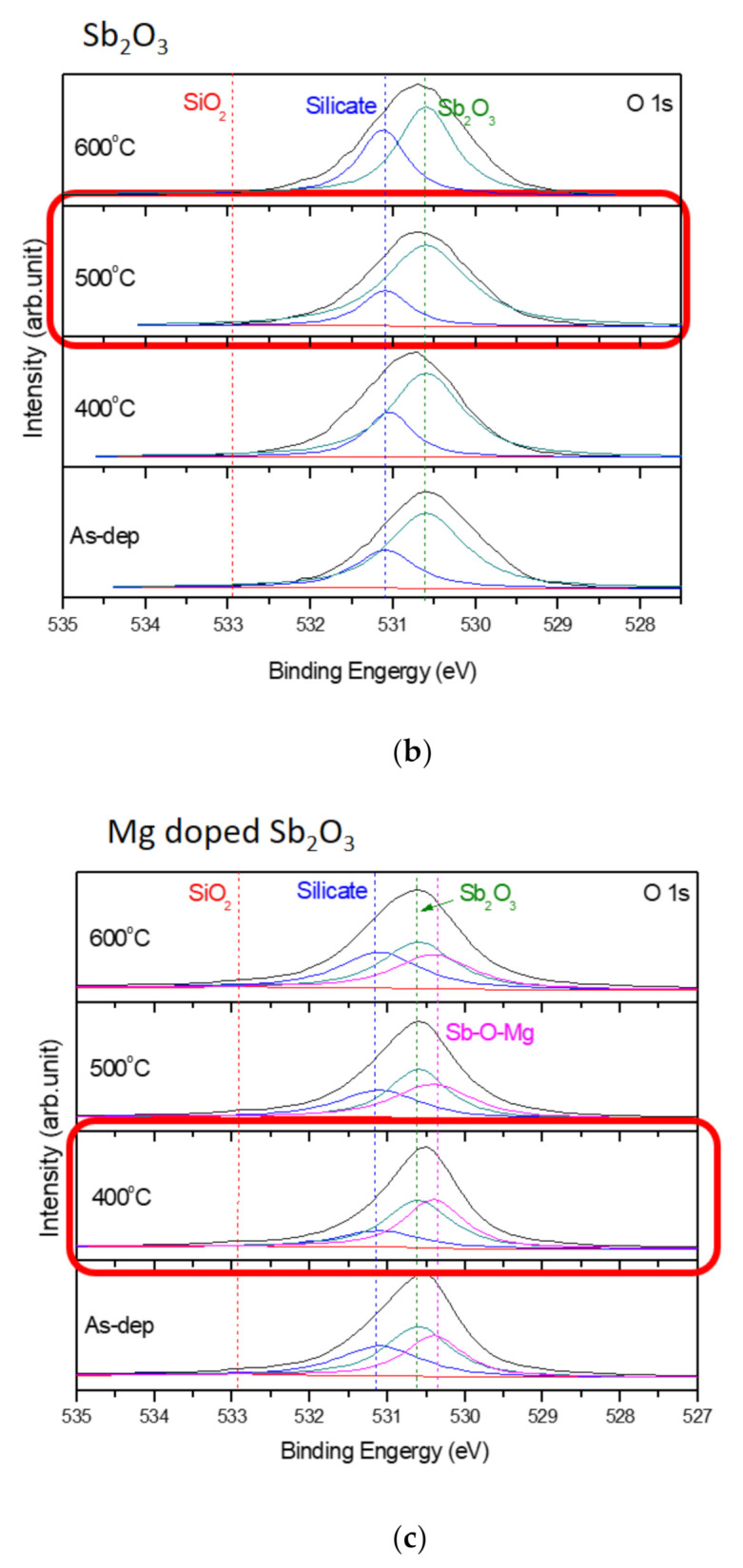

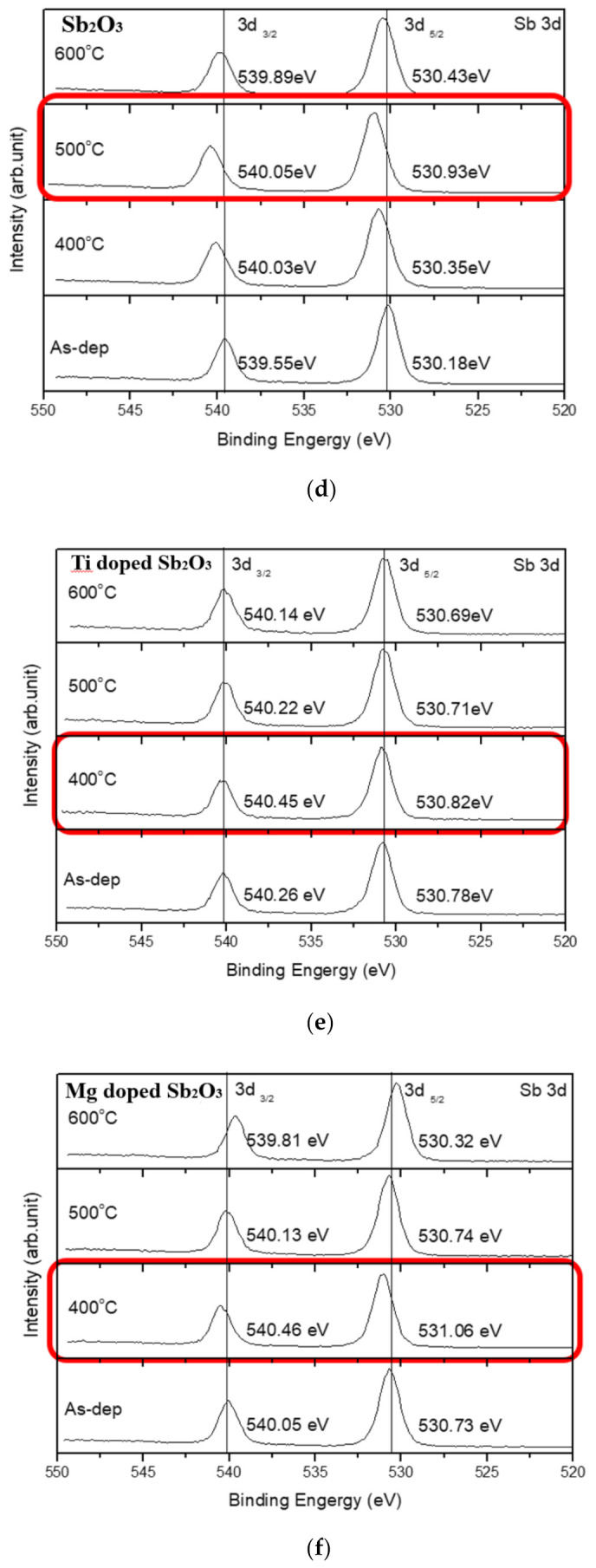

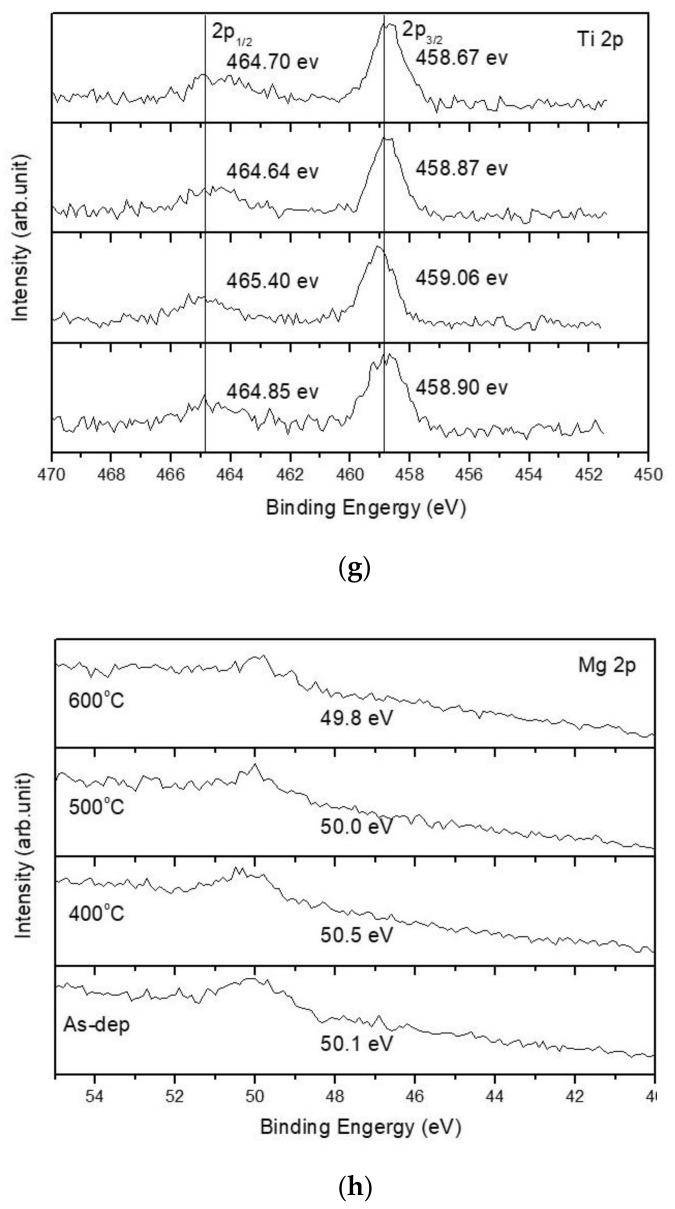
The O 1s XPS spectra of (**a**) the undoped Sb_2_O_3_ film, (**b**) the Ti-doped Sb_2_O_3_ films and (**c**) the Mg-doped Sb_2_O_3_ films. The Sb 3d XPS spectra of (**d**) the undoped Sb_2_O_3_ film, (**e**) the Ti-doped Sb_2_O_3_ films and (**f**) the Mg-doped Sb_2_O_3_ films annealed at various temperatures in O_2_ ambient for 30 s. (**g**) The Ti 2p XPS spectra of the Ti doped Sb_2_O_3_ film. (**h**) The Mg 2p XPS spectra of the Mg doped Sb_2_O_3_ film.

Furthermore, to monitor the chemical bindings and element compositions, the O 1s XPS analysis was performed on the undoped, Ti-doped and Mg-doped samples, as shown in Figure 4a–c, respectively. Figure 4a reveals that an RTA at an appropriate temperature of 500 °C could effectively suppress the formation of silicate and optimize the sensing device’s performance. However, as the annealing temperature further increased to 600 °C, the amount of silicate increased again. Similarly, annealing at 400 °C could effectively suppress the formation of silicate, as shown in the XPS spectra of Ti-doped and Mg-doped samples, as shown in Figure 4b,c. Moreover, annealing could strengthen Sb-O-Ti bonds and Sb-O-Mg bonds in Ti-doped and Mg-doped samples, respectively. Furthermore, the Sb 3d XPS analysis was performed on the undoped, Ti doped and Mg doped samples, as shown in Figure 4d–f, respectively. The Sb-O doublet peaks can be observed on these spectra. The Sb 3d XPS spectra also show that the Sb-O bonds could be strengthened with an appropriate annealing temperature of 500 °C for the undoped sample, as shown in Figure 4d. In addition, Mg doping could further enhance the Sb-O bonds in terms of Sb-O doublet peaks compared with the undoped samples. Consistent with the XRD patterns, silicate could be mitigated and chemical bindings could be enhanced by annealing and Mg doping [57]. Since XPS could complement the XRD analysis and was the technique used to identify the presence of the Mg and Ti bindings, the measurements of the Ti 2p XPS spectra for Ti-doped samples, as shown in Figure 4g, and the Mg 2p spectra for Mg-doped samples, as shown in Figure 4h, were performed, respectively. The results indicate that the XPS peaks for Ti and Mg did not vary a lot in various annealing conditions, but the peaks did represent the presence of Mg and Ti binding.

**Figure 5:**
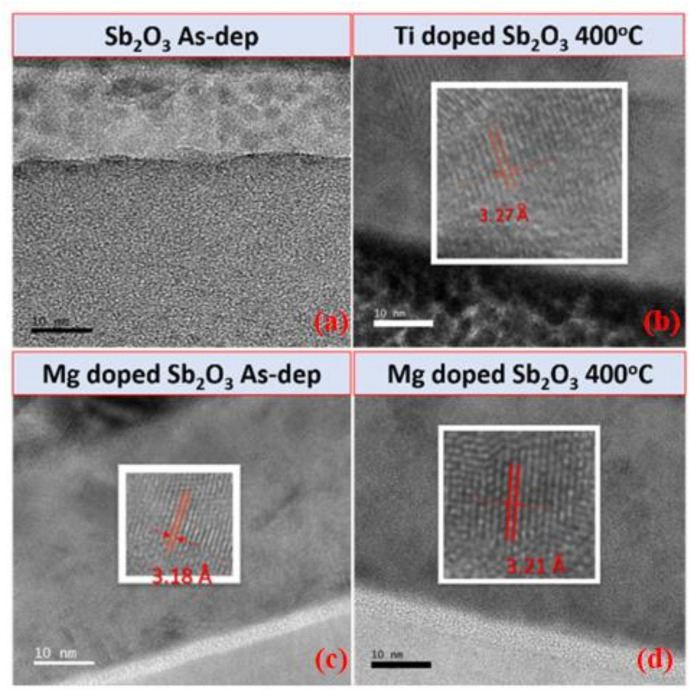
TEM and HRTEM images of (**a**) the as-deposited Sb_2_O_3_ films, (**b**) the Ti doped Sb_2_O_3_ film annealed at 400 °C, (**c**) the as-deposited Mg-doped Sb_2_O_3_ film and (**d**) the Mg-doped Sb_2_O_3_ film annealed at 400 °C.

To examine the nanostructures of the Sb_2_O_3_ samples, TEM and HRTEM were used to analyze the film in nanometer scale [58]. The TEM and HRTEM images of the as-deposited Sb_2_O_3_, the Ti-doped Sb_2_O_3_ sample annealed at 400 °C, the as-deposited Mg-doped Sb_2_O_3_ sample and the Mg-doped Sb_2_O_3_ sample annealed at 400 °C are shown in Figure 5a–d, respectively. The crystalline interval of the as-deposited sample is not clear. As for the annealed Ti-doped sample, the interval spacing can be observed on the TEM image, as shown in Figure 5b. In addition, the enlarged HRTEM image of the interval spacing reveals that the width interval spacing is 0.327 nm, which is close to the 0.322 nm of Sb_2_O_3_ (222) in a HRTEM image based on a previous report [59]. In addition, the interval spacing of the as-deposited Ti-doped sample can be seen in both the TEM and HRTEM images of Figure 5c. The morphology of the interval spacing of the annealed Ti-doped sample and the as-deposited Mg-doped sample are similar. Compared with the annealed Ti-doped sample and the as-deposited Mg-doped sample, the Mg-doped annealed sample exhibits clearer interval spacing in the TEM and HRTEM images, as shown in Figure 5d. Corresponding with the XRD patterns, as shown in Figure 2, the Mg-doped film annealed at 400 °C had the strongest Sb_2_O_3_ (222) phases, implying that the combination of Mg doping and annealing could optimize the film’s material quality.

**Figure 6:**
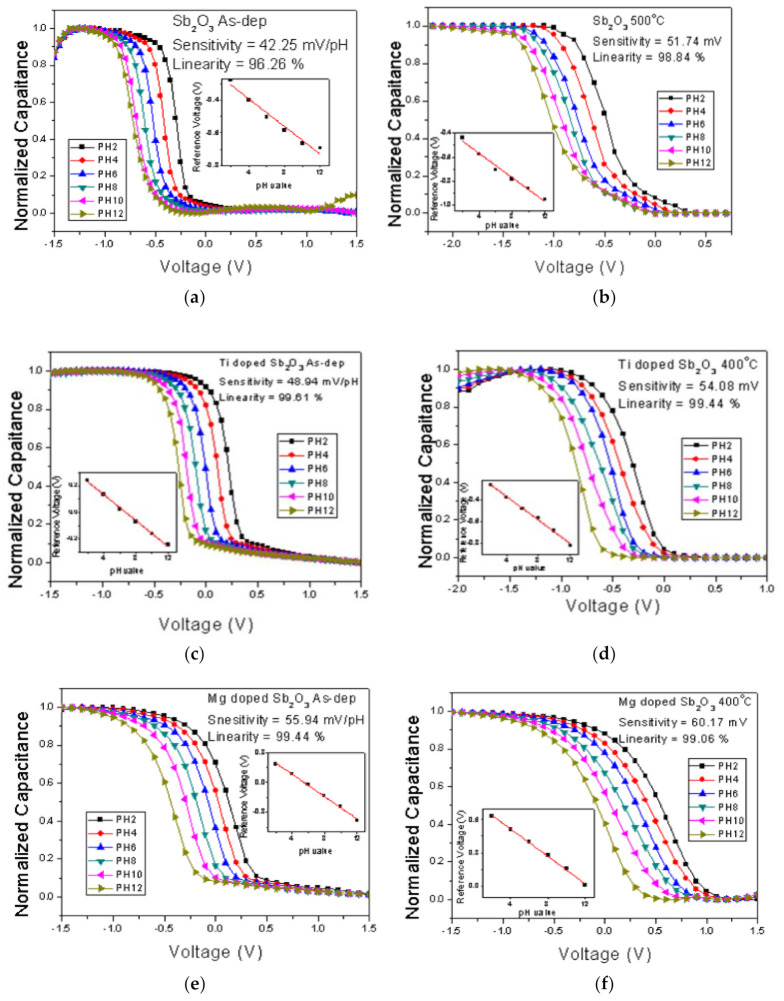
C–V curves of (**a**) the as-deposited Sb_2_O_3_ sensing membrane, (**b**) the Sb_2_O_3_ sensing membrane annealed at 500 °C, (**c**) the as-deposited Ti-doped Sb_2_O_3_ sensing membrane, (**d**) the Ti-doped Sb_2_O_3_ sensing membrane annealed at 400 °C, (**e**) the as-deposited Mg-doped sensing membrane and (**f**) the Mg-doped O_3_ sensing membrane annealed at 400 °C.

To measure the sensitivity and linearity of EIS capacitors, a Ketheley 2400 Source Meter was used to evaluate the C-V curves of the samples treated in various conditions. With 0.4 C_max_ set as the reference capacitance, the sensitivity and linearity could be calculated by extracting the points of various pH values with this reference capacitance. Figure 6a–f show the C-V curves of the EIS capacitors with the as-deposited Sb_2_O_3_ film and the Sb_2_O_3_ film annealed at 500 °C, the as-deposited Ti-doped Sb_2_O_3_ film, the Ti-doped Sb_2_O_3_ film annealed at 400 °C, the as-deposited Mg-doped film and the Mg-doped Sb_2_O_3_ film annealed at 400 °C, respectively.

**Figure 7:**
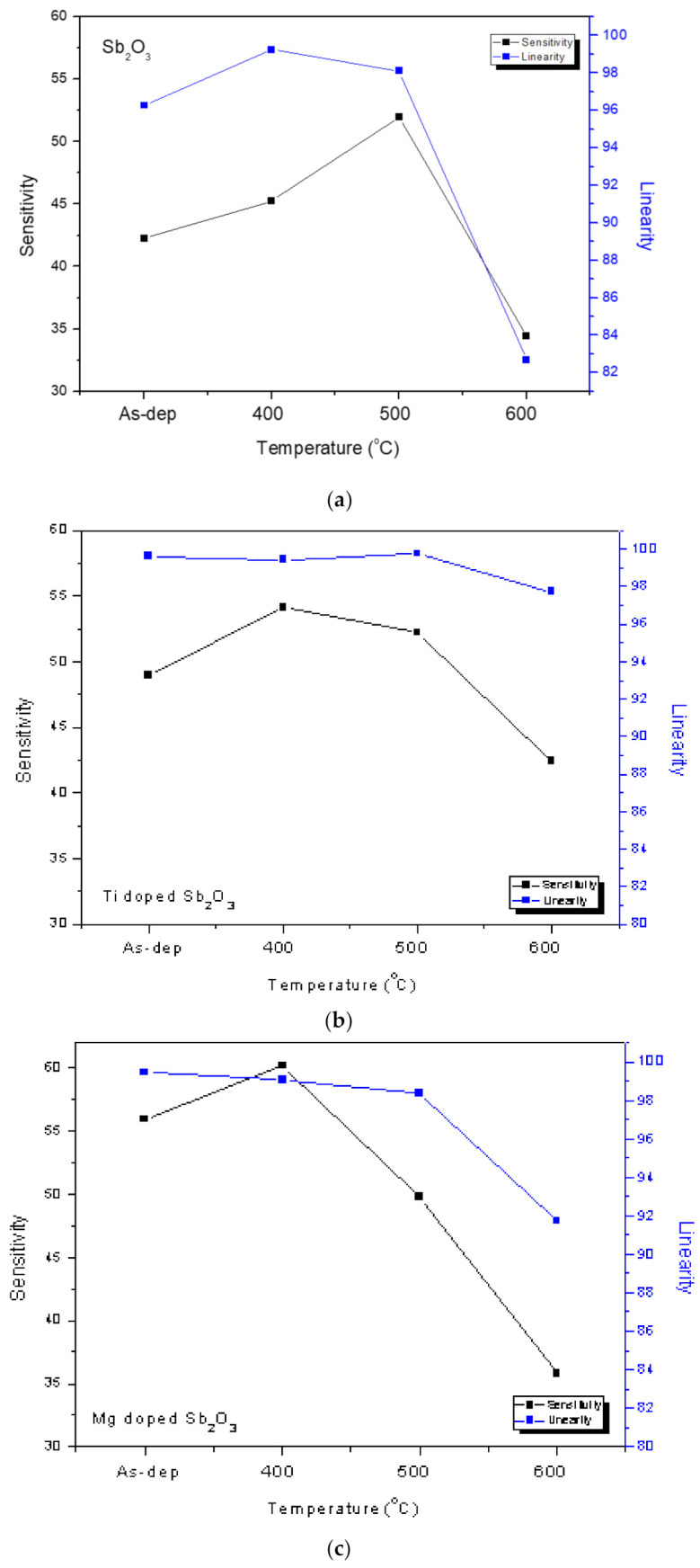
The pH sensitivity and linearity of (**a**) the undoped Sb_2_O_3_ membrane, (**b**) the Ti-doped Sb_2_O_3_ membrane and (**c**) the Mg-doped Sb_2_O_3_ membranes treated with different RTA temperatures in O_2_ ambient.

Consistent with the material characterizations, the pH sensitivity and linearity of the as-deposited membrane could be improved by annealing at 500 °C, as shown in Figure 6a,b. Moreover, the sensitivity and linearity could also be boosted by Ti doping and Mg doping without annealing, as shown in Figure 6c,e. In addition, appropriate annealing at 500 °C could further enhance the sensitivity of the Ti-doped samples from 48.94 to 54.08 mV/pH and the Mg-doped samples from 55.94 to 60.17 mV/pH, respectively, as shown in Figure 6d,f. All of the linearity and sensitivity data of the undoped, the Ti-doped and the Mg-doped samples treated with various annealing conditions are presented in Figure 7a–c.

**Figure 8:**
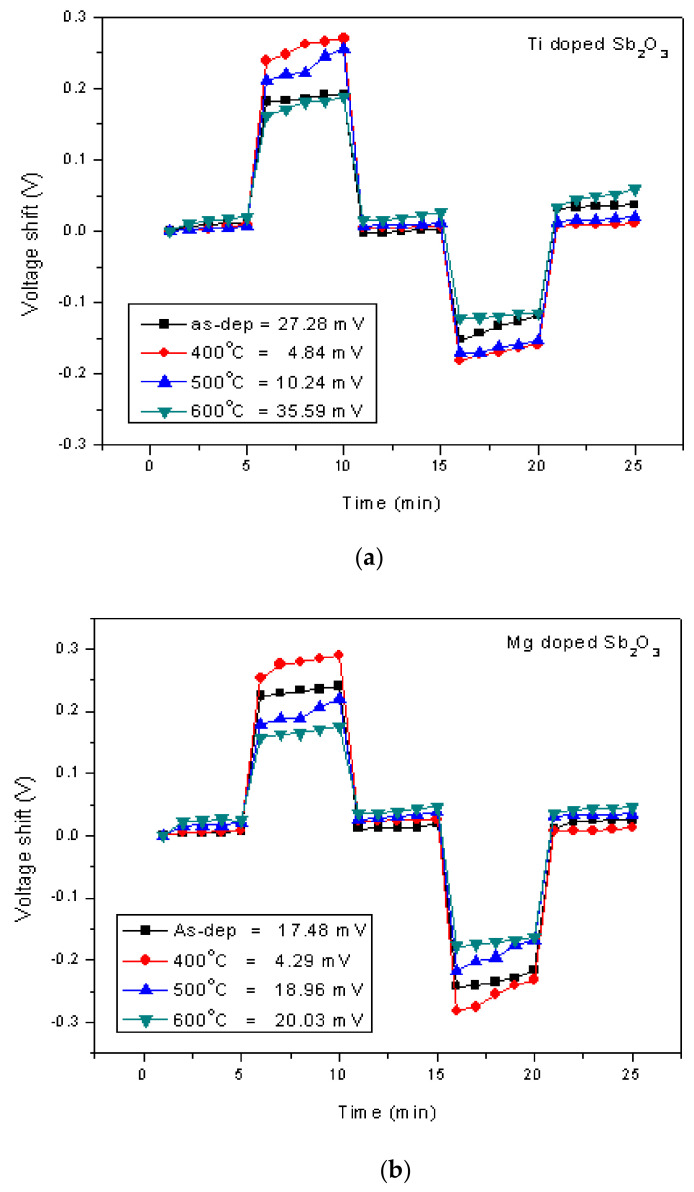

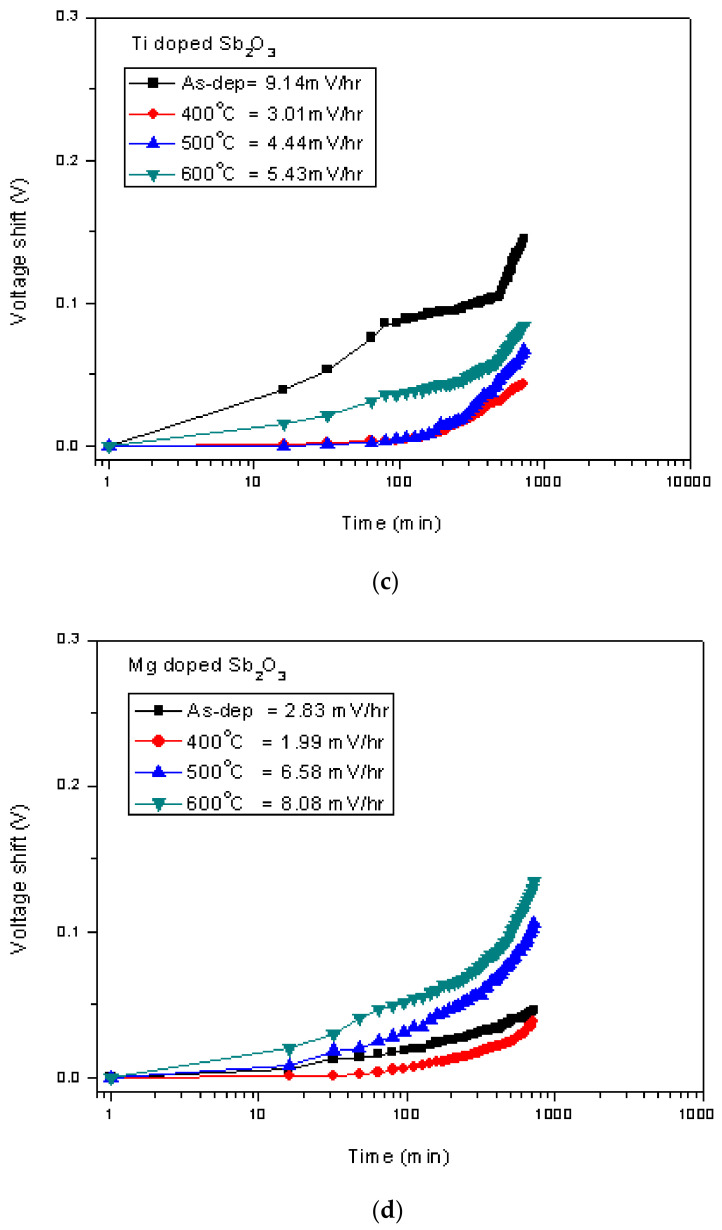
The hysteresis of (**a**) the Ti-doped Sb_2_O_3_ EIS membranes and (**b**) the Mg-doped Sb_2_O_3_ EIS membranes annealed in different conditions during a pH loop of 7→4→7→10. The drift voltage measurements of (**c**) the Ti-doped Sb_2_O_3_ EIS membranes and (**d**) the Mg-doped Sb_2_O_3_ EIS membranes annealed in different conditions.

As for the undoped samples, annealing at the appropriate temperatures of 400 °C and 500 °C effectively enhanced the sensitivity and linearity of the membrane, as shown in Figure 8a. Moreover, as shown in Figure 7b,c, in line with all of the material analyses, the combination of doping and appropriate annealing at 400 °C superpositionally increased both sensitivity and linearity for the Ti-doped and Mg-doped samples. Annealing with a high annealing temperature of 600 °C would degrade the material quality and the sensing behaviors of the doped Sb_2_O_3_ membranes. Material quality improvements enhanced the device sensing properties. Among all of the membranes, the Mg-doped membrane annealed at 400 °C with the strongest crystallization, grainization, and chemical bindings had the best pH sensitivity of 60.17 mV/pH and a high linearity of 99.06% [60,61].

To study stability and long-term reliability issues, the hysteresis voltages and the drift voltages were assessed. To calculate the hysteresis voltages, the samples were immersed in solutions with various pH values of 7, 4, 7, 10 in an alternating cycle with an immersion time of 5 min. The hysteresis voltage of the Ti-doped and Mg-doped samples with various annealing treatments are shown in Figure 8a,b, respectively. Consistent with the material characterizations and sensing measurements, the hysteresis voltages for the Ti-doped sample and the Mg-doped sample annealed at 400 °C had the lowest values of 27.28, 4.84 and 4.29 mV, respectively. Mg doping incorporated with annealing at 400 °C had the most reliable response. Since the dangling bonds and traps might capture H^+^ or OH^-^ ions in solutions, a membrane of better material quality might exhibit lower hysteresis voltages. Furthermore, to evaluate the capacitor’s long-term reliability, all of the tested samples were immersed in pH7 buffer solutions for 12 h, and the drift voltages were calculated. Similarly, the hysteresis voltages for the Ti-doped sample and the Mg-doped sample annealed at 400 °C had the smallest values of 3.01 and 1.99 mV/hr, respectively, as shown in Figure 8c,d. Since post-annealing at 400 °C incorporating Mg doping could effectively reduce vacancies and defects, the drift voltage of the Mg-doped sample annealed at 400 °C was greatly suppressed. These reliability tests were consistent with all of the other electrical measurements and material analyses [62].

**Figure 9:**
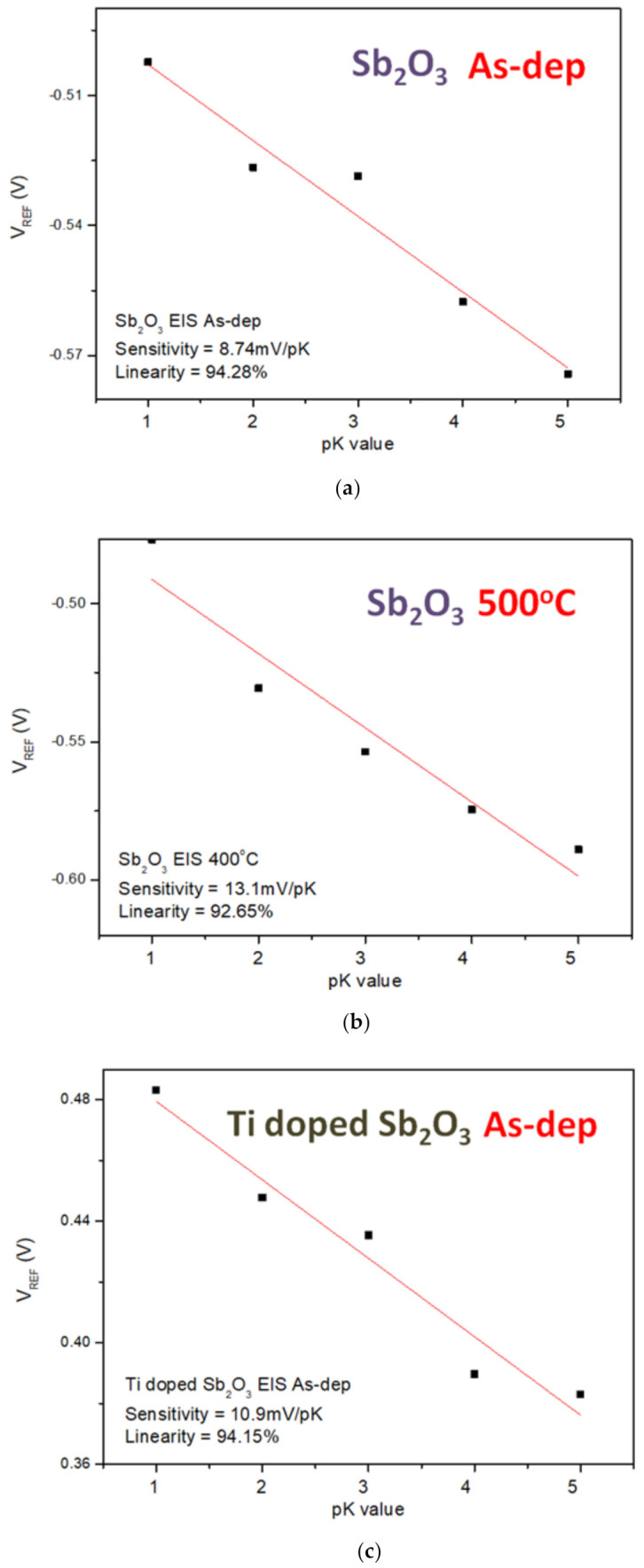

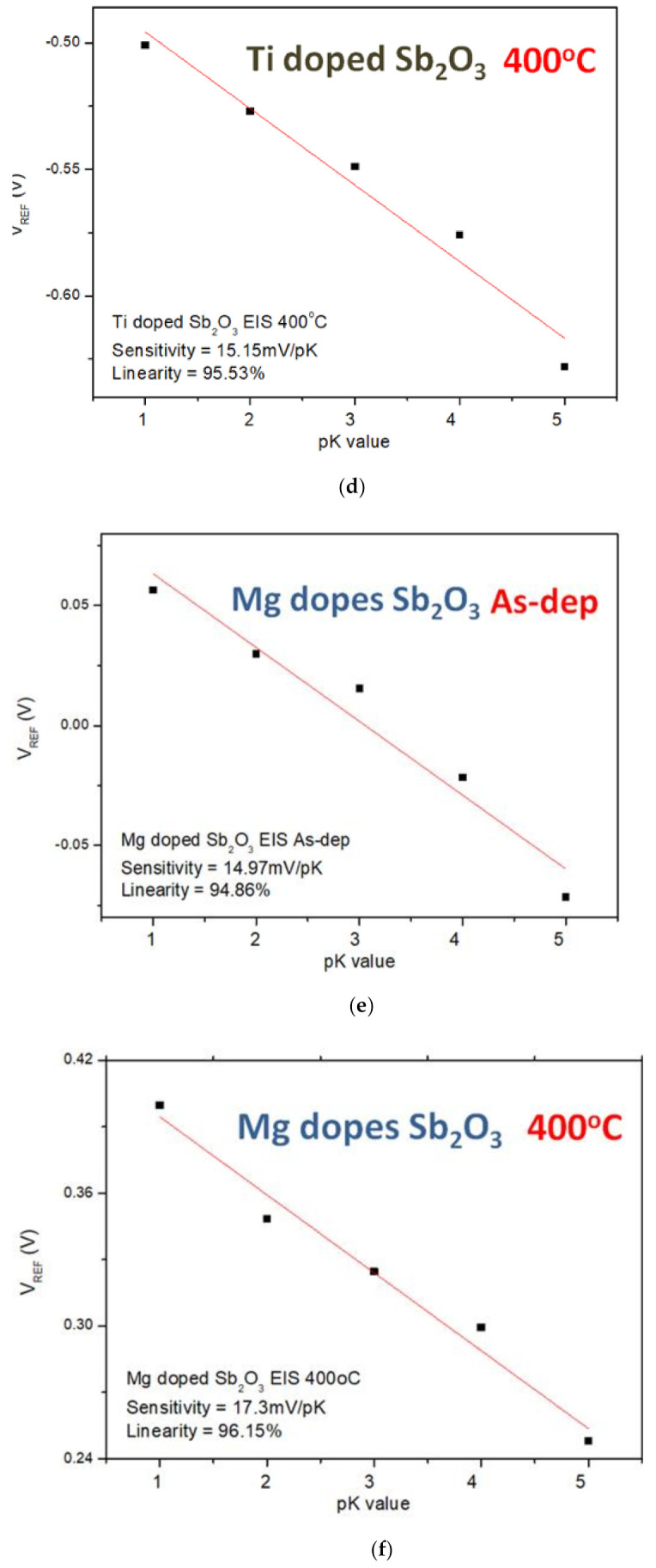
The K^+^ sensing behaviors of (**a**) the as-deposited Sb_2_O_3_ sensing membrane, (**b**) the Sb_2_O_3_ sensing membrane annealed at 500 °C, (**c**) the as-deposited Ti-doped Sb_2_O_3_ sensing membrane, (**d**) the Ti-doped Sb_2_O_3_ sensing membrane annealed at 400 °C, (**e**) the as-deposited Mg-doped sensing membrane and (**f**) the Mg-doped O_3_ sensing membrane annealed at 400 °C.

Finally, to compare the sensing sensitivity of different ions on the EIS structure, the sensitivity and linearity of the K^+^ and Na+ ions of the undoped, the Ti-doped and Mg-doped samples were measured. As shown in Figure 9a–f, the sensitivity and linearity measurement of K^+^ ion for the samples reveal that the Mg-doped sample annealed at 400 °C had the optimized sensitivity of 17.3 mV/pK and linearity of 96.15%.

**Figure 10:**
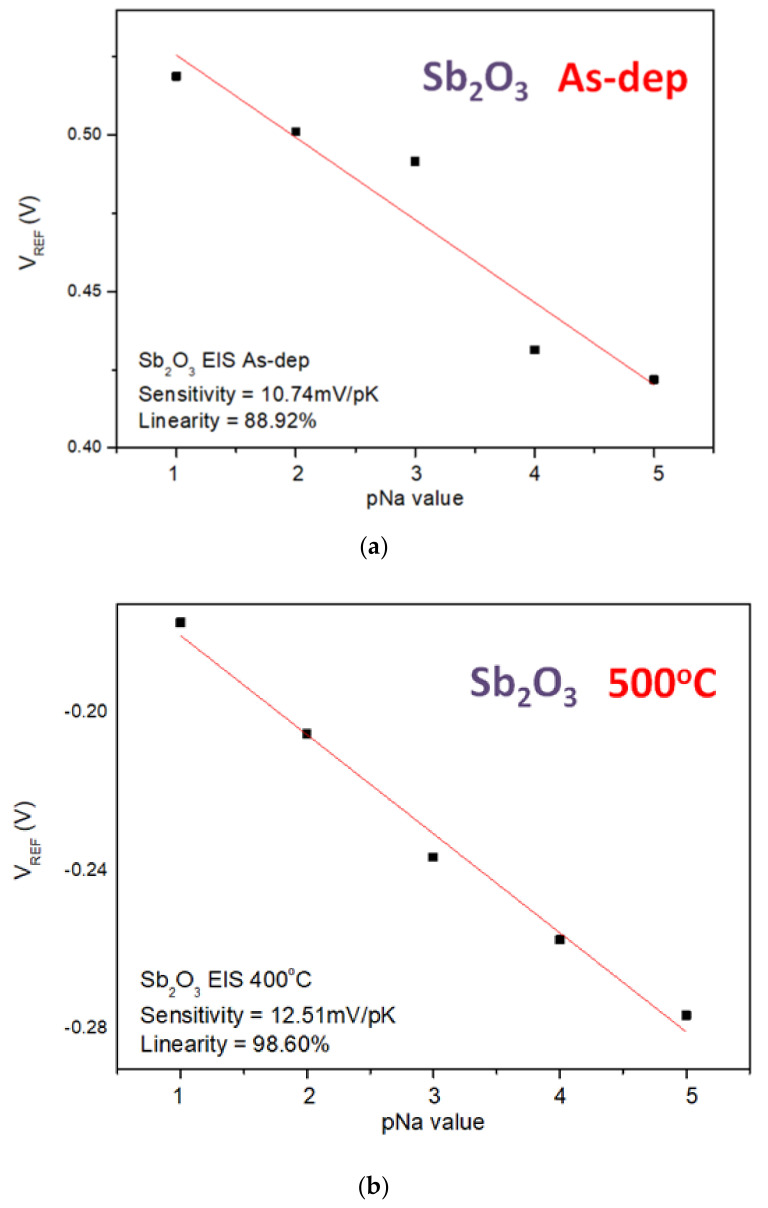

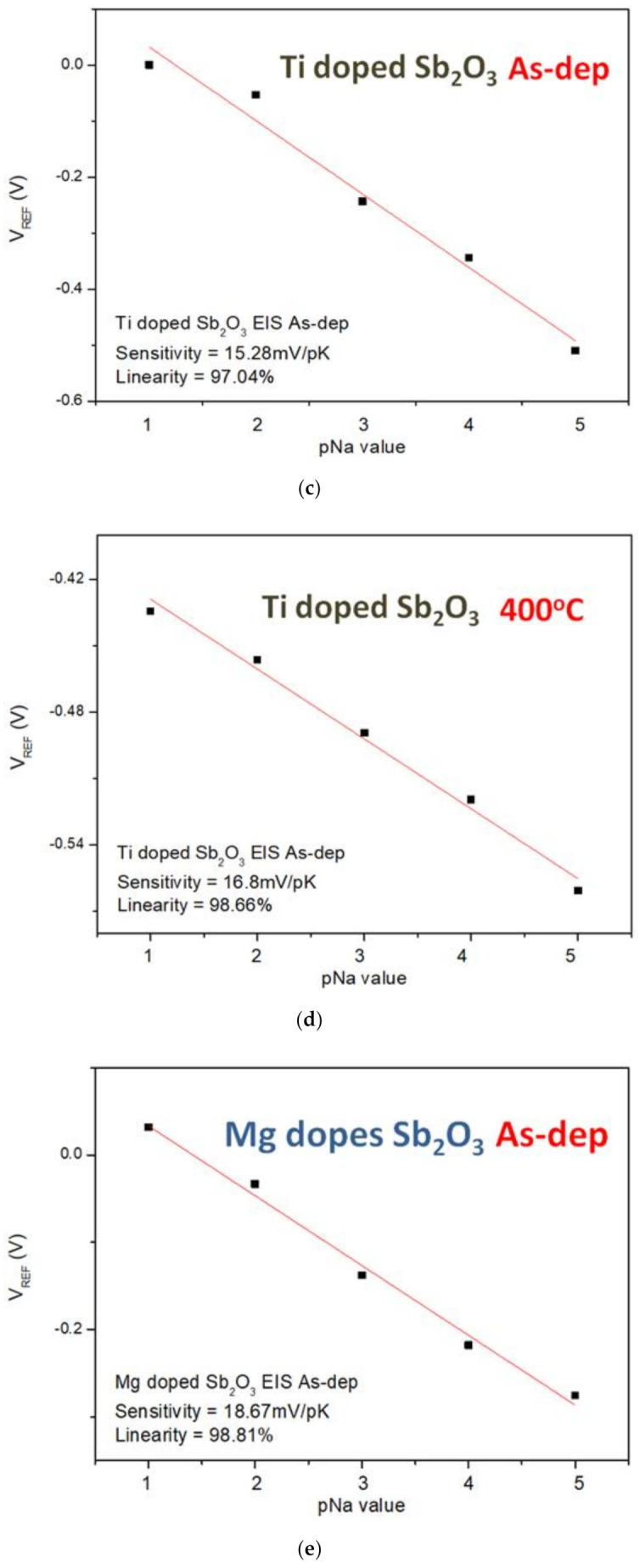

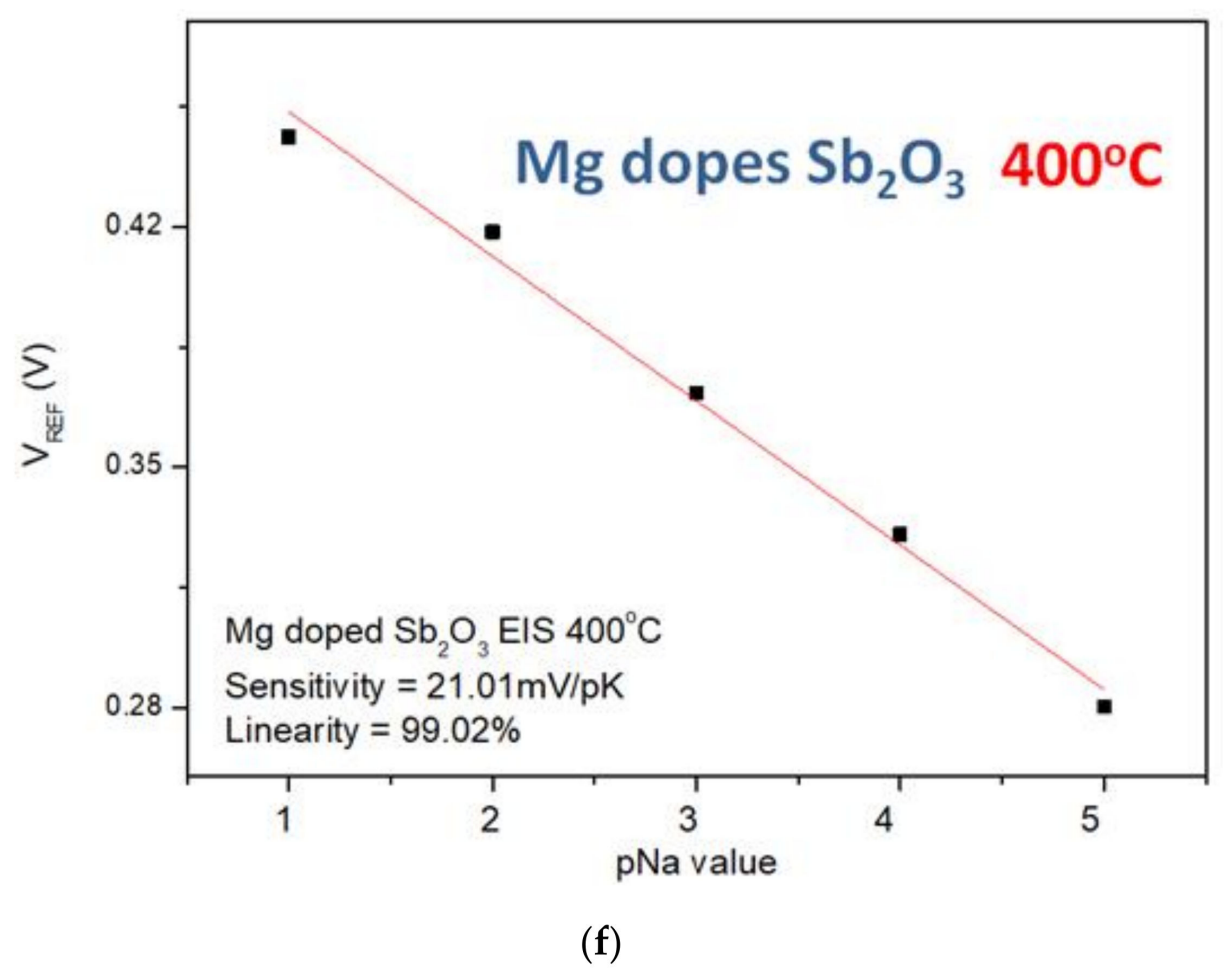
The Na^+^ sensing behaviors of (**a**) the as-deposited Sb_2_O_3_ sensing membrane, (**b**) the Sb_2_O_3_ sensing membrane annealed at 500 °C, (**c**) the as-deposited Ti-doped Sb_2_O_3_ sensing membrane, (**d**) the Ti-doped Sb_2_O_3_ sensing membrane annealed at 400 °C, (**e**) the as-deposited Mg-doped sensing membrane and (**f**) the Mg-doped O_3_ sensing membrane annealed at 400 °C.

Similarly, the sensitivity and linearity measurements of the Na^+^ ion for the samples reveal that the Mg-doped sample annealed at 400 °C had an optimized sensitivity of 21.01 mV/pK and a linearity of 96.15%, as shown in Figure 10a–f. Since the radius and mass of H^+^ ions are much smaller than the radius and mass of K^+^ and Na^+^, the sensitivity of the K^+^ and Na^+^ ions is smaller than that of the H^+^ ions.

**Figure 11:**
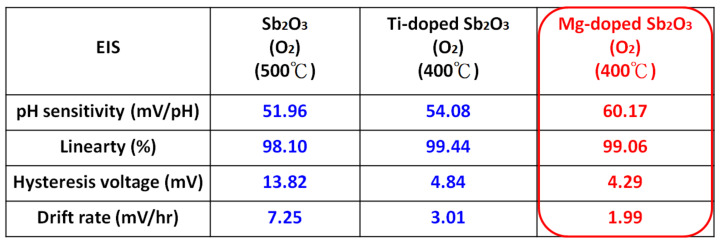
Comparison of the sensing behaviors of the appropriately annealed, undoped, Ti-doped and Mg-doped Sb_2_O_3_ membranes.

Finally, the sensitivity, linearity, hysteresis characteristics and drift characteristics of the appropriately annealed undoped, Ti-doped and Mg-doped Sb_2_O_3_ membranes were compared. The Mg-doped Sb_2_O_3_ membranes annealed at 400 °C still had the most preferable sensing characteristics compared with all of the other samples, as shown in Figure 11.

## 4. Conclusions

In this study, undoped, Ti-doped and Mg-doped Sb_2_O_3_ sensing membranes with various annealing conditions were fabricated. Multiple material characterizations and sensing measurements were conducted to study the annealing and doping effects on the membranes. The results indicate that Mg doping incorporating annealing at an appropriate temperature of 400 °C could optimize the material quality and enhance the sensing behaviors due to the suppression of silicate, the enhancement of crystallization, the boosting of sensing factors and the removal of defects. Mg-doped Sb_2_O_3_-based membranes with appropriate annealing are promising for future industrial ion-sensing devices and for possible integration with Sb_2_O_3_-based devices.

## Data Availability

The data used to support the findings of this study are available from the corresponding author upon request.
